# Sex- and Stage-Specific Predictors of Anemia in Chronic Kidney Disease: A Retrospective Cohort Study

**DOI:** 10.3390/jcm14093088

**Published:** 2025-04-29

**Authors:** Jui-Ting Chang, Chun-Ji Lin, Jiann-Horng Yeh, Chin-Hung Tsai, I-Shan Hsieh, Po-Ya Chang

**Affiliations:** 1 School of Medicine, College of Medicine, Fu Jen Catholic University, Taipei 242, Taiwan; 2 Division of Nephrology, Department of Internal Medicine, Shin Kong Wu Ho-Su Memorial Hospital, Taipei 111, Taiwan; 3 Nutrition Department, Shin Kong Wu Ho-Su Memorial Hospital, No. 95, Wen Chang Road, Shih Lin District, Taipei 111, Taiwan; 4 Institute of Population Health Sciences, National Health Research Institutes, Miaoli County 350, Taiwan; 5 Department of Neurology, Shin Kong Wu Ho-Su Memorial Hospital, Taipei 111, Taiwan; 6 Department of Neurology, Kaohsiung Medical University, Kaohsiung 807, Taiwan; 7 Department of Chest Medicine, Tungs’ Taichung MetroHarbor Hospital, Taichung 435, Taiwan; 8 Cancer Center, Tungs’ Taichung MetroHarbor Hospital, Taichung 435, Taiwan; 9 Department of Post-Baccalaureate Medicine, College of Medicine, National Chung Hsing University, Taichung 402, Taiwan; 10 Department of Leisure Industry and Health Promotion, National Taipei University of Nursing and Health Sciences, No. 365, Ming-te Road, Peitou District, Taipei 112, Taiwan

**Keywords:** anemia, chronic kidney disease, risk factor, predictor, cohort study

## Abstract

**Background:** Anemia is a common complication of chronic kidney disease (CKD), yet no study has explored differences in anemia risk factors based on disease severity and gender. Therefore, this study investigates potential differences in anemia risk among individuals with varied kidney disease severities and sexes. **Methods:** This multicenter, longitudinal cohort study was conducted using data (2008–2016) from the Epidemiology and Risk Factors Surveillance of CKD database. This database was associated with Taiwan’s National Health Insurance Research Database (for the 2008–2019 period). To identify predictive risk factors for anemia, we developed a subset multivariate logistic model using stepwise variable selection. Additionally, 10-fold cross-validation was conducted to facilitate model selection and internal validation. **Results:** Of the 5656 patients with CKD, 519 (9.18%) with anemia and 5137 (90.82%) without. After adjusting for age, sex, and serum creatinine, stepwise logistic regression analysis identified the main independent predictive factors for anemia in CKD patients. Notably, “Receive low sodium diet education” (OR: 0.66, 95% CI: 0.446–0.975), “DBP (mmHg)” (OR: 0.98, 95% CI: 0.965–0.999), “Gout” (OR: 1.86, 95% CI: 1.175–2.937), and “Congestive heart failure” (OR: 1.85, 95% CI: 1.131–3.028) was significantly associated with the presence of anemia among CKD patients. **Conclusions:** This study identifies gout and cardiovascular disease as important correlates of anemia in patients with CKD. Moreover, it reveals an inverse association between elevated diastolic blood pressure and receiving education on a low-sodium diet with the occurrence of anemia.

## 1. Introduction

Chronic kidney disease (CKD) is an important public health issue worldwide that significantly contributes to morbidity and mortality [1]. Anemia is a common complication of CKD and affects approximately 15% of patients with this disease [2]. As kidney function progressively deteriorates in CKD, anemia tends to worsen [2,3]. According to data from the Japan Chronic Kidney Disease Database, the prevalence of anemia in patients with Stage 4 CKD is 40.1%, whereas that in patients with Stage 5 CKD is 60.3% [4].

In a previous cohort study by the US Veterans Administration that involved 933,463 patients with CKD, 20.6% had anemia. Additionally, absolute and functional iron deficiency anemia were associated with various clinical covariates [5]. Patients with CKD plus anemia who are not receiving effective treatment are at increased risk of other comorbidities, including cognitive impairment, sleep disorder, renal progression, cardiovascular disease, and mortality [6,7,8]. Epidemiologic studies have provided evidence indicating that lower hemoglobin (Hb) levels are associated with an increased risk of requiring subsequent kidney replacement therapy and of experiencing heart failure, stroke, coronary heart disease, or death [9].

Erythropoietin (EPO) deficiency contributes to the development of anemia in CKD [10]. Patients experiencing anemia with preserved kidney function exhibit EPO levels that are 10 to 100 times greater than those observed in patients with CKD-associated anemia [11,12,13]. Insufficient renal EPO production is the principal causative factor of anemia in patients with CKD. This insufficiency arises from complex interactions involving patient-specific attributes [14].

A cross-sectional study in China revealed that the prevalence of anemia in patients with diabetic nephropathy was higher than that in patients with kidney damage caused by chronic hypertension or chronic glomerulonephritis [15]. Additionally, a study conducted in South Africa revealed an association of CKD with diabetes mellitus and anemia [16]. The presence of advanced CKD, hematological disorders, respiratory disorders [17], rural residence, a body mass index (BMI) < 18.5 kg/m^2^, and a BMI of 18.5–24.9 kg/m^2^ [18] are associated with an increased risk of developing CKD-associated anemia. Conversely, a higher previous Hb concentration and the use of iron supplements can reduce the risk of developing anemia in patients with CKD [17].

Several studies have investigated the risk factors associated with anemia in CKD [14,19]. Notably, the prevalence and severity of anemia are related to sex and the degree of kidney function deterioration, respectively [9]. Research findings indicate that a decreased estimated glomerular filtration rate (eGFR) is associated with a higher prevalence of anemia in CKD [9]. Furthermore, under different kidney function conditions, the prevalence of anemia in women surpasses that in men [9]. However, while these studies have identified broad trends, they have not thoroughly explored the stratified risk profiles—that is, how specific anemia-associated factors may differ by sex or by CKD severity. For instance, it remains unclear whether certain clinical or lifestyle factors (e.g., gout, HbA1c, blood pressure) are more relevant in particular subgroups. Therefore, the current multicenter cohort study linked claims databases to investigate the risk factors for anemia in patients with CKD. Additionally, the study explored potential differences in the risk factors for anemia for individuals with different kidney disease severities and sexes.

## 2. Materials and Methods

### 2.1. Study Design and Participants

This was a prospective study using data from the Epidemiology and Risk Factors Surveillance Program (2008–2016) and Taiwan’s National Health Insurance Research Database Ministry of Health and Welfare (NHIRD_MOHW) (2008–2021).

The study involved patients with CKD from 14 hospitals and communities across Taiwan, covering the northern, central, and southern regions. These institutions comprised Taipei Veterans General Hospital, Taipei Medical University Hospital, Tri-Service General Hospital, Taipei Chang Gung Memorial Hospital (Chang Gung Medical Foundation), Shuang Ho Hospital, Show Chwan Memorial Hospital, Changhua Christian Hospital, China Medical University Hospital, National Cheng Kung University Hospital, Kaohsiung Chang Gung Memorial Hospital, E-DA Hospital, Kaohsiung Municipal Siaogang Hospital, and Kaohsiung Medical University Chung-Ho Memorial Hospital. An initial sample of 16,470 participants was obtained. Patients with incomplete kidney function data (*n* = 88), follow-up durations of less than 12 months (*n* = 1877), a single serum creatinine (SCr) measurement (*n* = 6243), and without CKD (*n* = 2606) were excluded from the study. Ultimately, the study included 5656 patients.

CKD was defined according to the Kidney Disease Outcomes Quality Initiative guidelines [20]. The estimated glomerular filtration rate (eGFR) was calculated using the Chronic Kidney Epidemiology Collaboration (CKD-EPI) Taiwan equation: 1.262 × [141 × min(SCr)/κ,1)^α^ × max(SCr/κ,1)^−1.209^ × 0.993^age^ × 1.018 (if female) × 1.159 (if black)]^0.914^, where κ is 0.7 for females and 0.9 for males, α is −0.329 for females and −0.411 for males, and min and max denote the minimum and maximum of SCr/κ or 1, respectively [21,22]. CKD was categorized into early stages (stage 1, stage 2, and stage 3a) and late stages (stage 3b, stage 4, and stage 5) based on the severity of kidney damage. Stage 1 represents kidney damage detectable by proteinuria dipsticks ≥ 1+, urine protein-to-creatinine ratio (UPCR) ≥ 150, or urine albumin-to-creatinine ratio (UACR) ≥ 30, with eGFR ≥ 90 mL/min/1.73 m^2^. Stage 2: indicates kidney damage as mentioned above, with an eGFR of 60–89 mL/min/1.73 m^2^. Stage 3a is defined by an eGFR of 45–59 mL/min/1.73 m^2^. Stage 3b is defined by an eGFR of 30–44 mL/min/1.73 m^2^. Stage 4 represents an eGFR of 15–29 mL/min/1.73 m^2^, and Stage 5 indicates an eGFR of less than 15 mL/min/1.73 m^2^ [23]. The distribution of patients across CKD stages was as follows: Stage 1: 306 (5.41%), Stage 2: 1427 (25.23%), Stage 3a: 1092 (19.31%), Stage 3b: 993 (17.56%), Stage 4: 1125 (19.89%), and Stage 5: 713 (12.61%).

We used the patients’ unique personal identification numbers to link their Epidemiology and Risk Factors Surveillance data with their medical records, which were retrieved from the NHIRD_MOHW. The NHIRD_MOHW is a comprehensive, population-based insurance claims database that includes the data of more than 99% of Taiwan’s residents [24]. This database serves as a rich repository of medical and pharmacy claims data and contains records of diagnoses and healthcare service utilization [24]. We obtained the following data from the NHIRD_MOHW: diagnosis of anemia and medications for anemia treatment.

### 2.2. Study Variables and Definitions

The participants self-reported their demographic characteristics, including their sex, age, and retirement status, as well as their health-related behaviors, such as their smoking status and alcohol consumption. Cigarette smoking was defined as having a history of smoking at least 100 cigarettes in one’s lifetime [25]. Alcohol consumption was defined as regular consumption of alcoholic beverages [26]. Furthermore, participants indicated whether they had received dietary and health education, including nutrition education, low-sodium diet education, low phosphorus diet education, and low-protein diet education.

All participating hospitals and communities adhered to a uniform set of medical laboratory standards and protocols, which enabled extraction and comparison of the following clinical and laboratory data from the patients’ medical records: height, weight, waist circumference, BMI, SCr level, baseline eGFR, fasting glucose level, systolic blood pressure (SBP), diastolic blood pressure (DBP), uric acid, blood urea nitrogen level (BUN), total cholesterol, triglyceride level, sodium (Na) level, phosphorus (P) level, glycated hemoglobin (HbA1c) level, proteinuria, and Hb level.

Anemia and chronic diseases were identified using the International Classification of Diseases, Ninth, and Tenth Revision, Clinical Modification (ICD-9-CM and ICD-10-CM) codes. Anemia was defined as having at least two outpatient or inpatient records of anemia diagnosis within one year. The use of erythropoietin (EPO) or iron supplements, identified through Anatomical Therapeutic Chemical (ATC) codes, was also considered as part of anemia management. Comorbidities included diabetes, hypertension, dyslipidemia, gout, cerebrovascular disease, ischemic heart disease, congestive heart failure, urinary tract infection, depression, and cancer. Detailed ICD and ATC codes for each condition are provided in Supplementary Table S1. This study was conducted in accordance with the ethical principles of the Declaration of Helsinki and was approved by the Institutional Review Board of Shin Kong Wu Ho-Su Memorial Hospital (IRB Number: 20230608R).

### 2.3. Statistical Analysis

The baseline characteristics were compared between the patients with and without anemia. The chi-square or Fisher’s exact test was used for between-group comparisons of categorical variables, and Student’s *t*-test was used for between-group comparisons of continuous variables. Logistic regression was used to assess the risk of anemia on the basis of demographic characteristics, health-related behaviors, dietary and health education, hematological and biochemical indicators, comorbidities, and other factors. After adjustment for age, sex, SCr, and hemoglobin, a stepwise selection method was used to identify significant predictors of anemia. Predictors were entered into the model with a significance level for entry (SLE) set at 0.10 and a significance level for stay (SLS) set at ≤ 0.05. Additionally, 10-fold cross-validation was conducted to facilitate model selection and internal validation. All statistical analyses were performed using SAS (version 9.4; SAS Institute, Cary, NC, USA).

## 3. Results

Table 1 describes the baseline characteristics of the study cohort. Of the 5656 patients with CKD (mean age: 62.57 ± 14.00; men, 58.38%), 519 (9.18%) with anemia and 5137 (90.82%) without. The comorbidities were hypertension (64.73%), diabetes mellitus (42.29%), dyslipidemia (24.66%), gout (17.63%), ischemic heart disease (23.97%), stroke (17.68%), congestive heart failure (8.42%), urinary tract infection (18.1%), depression (7.04%), and cancer (16.65%). Among patients with anemia, 26.19% did not use any medication. Among those who used medication, 18.88% utilized medicinal iron, 12.52% opted for erythropoiesis-stimulating agents, and 44.7% employed a combination of both medicinal iron and erythropoiesis-stimulating agents (Supplementary Table S2). In comparison to CKD patients without anemia, those with anemia were older, had lower eGFR, received more nutrition/diet education, and had a higher number of comorbidities. Supplementary Tables S3 and S4 present the baseline clinical characteristics of included patients based on sex and the classification of early and late CKD, respectively.

Univariate analyses were conducted to investigate the associations between various factors and anemia among patients with CKD (Supplementary Table S5). Age demonstrated a significant association with anemia. Sex differences were also observed, with a higher prevalence of CKD-associated anemia among women compared to men. Additionally, anemia was more prevalent in patients with advanced stages of CKD than in those with early-stage CKD. Retirement status, patients who received nutrition and diet education, and higher levels of BUN and SCr were associated with anemia. Among the assessed comorbidities, hypertension, gout, ischemic heart disease, stroke, congestive heart failure, urinary tract infection, depression, and cancer exhibited significant positive correlations with anemia in patients with CKD. Additionally, a waist circumference (odds ratio [OR]: 0.98, 95% confidence interval [CI]: 0.97–0.99), DBP (OR: 0.98, 95% confidence interval [CI]: 0.97–0.99), and BMI (OR: 0.92, 95% CI: 0.90–0.95) were negatively correlated with anemia.

Table 2 presents the predictive risk factors associated with anemia among patients with CKD. These factors were identified using univariate analyses (Supplementary Table S5) and were subsequently included in a multivariate logistic regression model by using stepwise selection. The model was adjusted for age, sex, SCr, and hemoglobin levels. Receiving a low-sodium diet education (OR: 0.66, 95% CI: 0.446–0.975), DBP (OR: 0.98, 95% CI: 0.965–0.999), gout (OR: 1.86, 95% CI: 1.175–2.937), and congestive heart failure (OR: 1.85, 95% CI: 1.131–3.028) were significant predictors of anemia among patients with CKD (C statistic = 0.78). Furthermore, after being subjected to 10-fold cross-validation, the model achieved a remarkable accuracy rate of 0.92.

**Figure 1:**
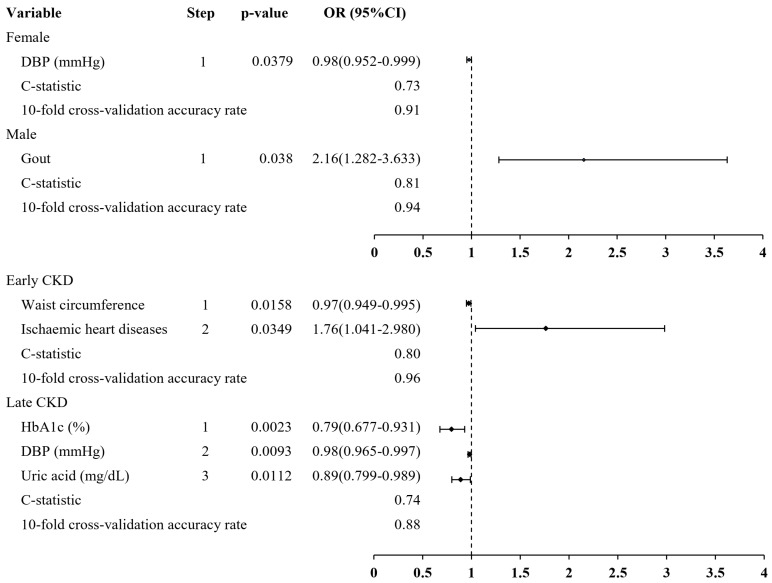
Predictive factors associated with anemia by sex and severity of kidney function among CKD patients.

Figure 1 presents the results of stratified analyses by sex and CKD stage for predicting the risk of anemia among patients with CKD. After adjusting for age, SCr, and hemoglobin levels, stepwise logistic regression analysis revealed that the main independent predictive risk factor for the female CKD patient group was DBP (OR: 0.98, 95% CI: 0.952–0.999), exhibiting a significant association with anemia (C statistic = 0.73). The model, after undergoing 10-fold cross-validation, demonstrated a notable accuracy rate of 0.91. Among the male patients, gout (OR: 2.16, 95% CI: 1.282–3.633) exhibited a significant positive association with anemia in this group (C statistic = 0.81). The accuracy rate, determined through 10-fold cross-validation, was 0.94. Within the group of patients with early-stage CKD, waist circumference exhibited a significant association with anemia. Ischemic heart diseases (OR: 1.76, 95% CI: 1.041–2.980) also exhibited significant positive associations with anemia (C statistic = 0.80). The accuracy rate, determined through 10-fold cross-validation, was 0.96. Among the patients with late-stage CKD, HbA1c levels, uric acid levels, and DBP were significantly negatively correlated with anemia (C statistic = 0.74). After 10-fold cross-validation, the accuracy of the model was 0.88.

## 4. Discussion

This study investigated the risk factors for anemia in individuals with CKD. Furthermore, the study conducted a detailed analysis of the differences in the risk of anemia based on sex and the severity of CKD. This study identified both overall and subgroup-specific predictors of anemia in patients with CKD. At the population level, significant risk factors included gout and congestive heart failure. Notably, after stratifying by sex and CKD stage, we found that certain predictors—such as gout in men, HbA1c and uric acid levels in late-stage CKD, and waist circumference in early-stage CKD—were uniquely associated with anemia risk within specific subgroups. These findings provide new insight into how anemia risk may vary across clinically meaningful categories of CKD patients.

This study revealed some predictive risk factors to be associated with anemia. Notably, gout and cardiovascular diseases were the most significant risk factors for anemia in patients with CKD, which is consistent with the findings from previous studies. Hyperuricemia with gouty attacks is a common disease in patients with CKD [27]. We observed that the presence of gouty arthritis was significantly associated with higher odds of anemia. In a previous study, cytokines such as interleukin (IL)-16 and IL-18, which are common mediators of inflammation, exhibited strong associations with the severity of gout. The clinical manifestations of gouty arthritis include fluctuations in serum levels of uric acid, tophi formations, and articular deformities [28]. Furthermore, anemia can result from proinflammatory cytokines disrupting iron homeostasis and EPO synthesis, both of which are essential for erythropoiesis [29]. Therefore, the risk of anemia is higher in patients with kidney disease who also have gout. In clinical practice, anemia is commonly observed in patients with congestive heart failure. The confluence of anemia, congestive heart failure, and CKD gives rise to an intricate and deleterious interplay, recognized as cardio-renal anemia syndrome. The arrest of CKD and congestive heart failure progression necessitates the implementation of a comprehensive therapeutic strategy concurrently targeting all three conditions [30]. Congestive heart failure is associated with an elevated production of inflammatory cytokines, specifically TNF-α and IL-6. These cytokines are linked to inadequate endogenous erythropoietin production in response to anemia, suppressed erythropoietic response of red cell precursors, and hindered intestinal iron absorption [31,32].

The current study also identified an association between elevated DBP and receiving education regarding a low-sodium diet with a reduced likelihood of developing anemia. Idris et al. (2018) similarly indicated that elevated DBP (OR: 0.97, 95% CI: 0.95–0.99, *p* < 0.001) was linked to a reduced risk of anemia [33]. Yoon et al. (2018) [34] reported a positive relationship between anemia and SBP, along with an inverse correlation between anemia and DBP. This indicates that as Hb levels decrease, SBP tends to rise, whereas a reduction in Hb and hematocrits is associated with a decline in DBP [34]. One possible explanation for this relationship is the role of plasma volume (PV) expansion in CKD-related anemia. Lundby et al. (2018) demonstrated that in anemic CKD patients, RBC volume (RBCV) was often within the expected range, whereas plasma volume was significantly increased [35]. This suggests that anemia in CKD may not always result from a true reduction in RBC production but rather from hemodilution due to plasma expansion, which could also influence DBP [35]. Furthermore, previous studies have demonstrated an inverse relationship between plasma volume and DBP, suggesting that intravascular volume dynamics play a crucial role in blood pressure regulation [36]. This mechanism might explain the observed inverse association between DBP and anemia risk in CKD patients. Additionally, low-sodium diet education was reported to be an effective means of reducing anemia prevalence because it was associated with restricted dietary salt intake and mitigated sugar-induced chronic inflammation [37]. Especially, hyperglycemia-induced advanced glycation end product (AGE) is a strong inflammation mediator [38]. The related cytokines also decrease the expressions of erythropoietin receptor messenger ribonucleic acid contributing to the major cause of anemia [39].

Figure 1 illustrates the distinct patterns of anemia risk factors between male and female CKD patients, highlighting how variables such as DBP and gout exhibit sex-specific associations that may reflect biological or treatment-related differences. To women, we found a lower prevalence of anemia in female patients with CKD having higher DBP. In clinical practice, it is well-known that hypertension is an independent risk factor for cardiovascular disease in patients with renal disease [40]. Moreover, isolated hypertension with high pulse pressure (PP), calculated as the difference between SBP and DBP, has been associated with a higher risk of target organ damage in the same population [41]. However, there were a few studies mentioning the relationship among anemia, women, and DBP. Okada et al. reported a higher prevalence of anemia was observed in women with CKD compared to men, and slight decreases in Hb and mild anemia are independent predictors of decreasing eGFR specifically in women with early-stage CKD [42]. A higher prevalence of anemia in women with CKD may result from more iron deficiency anemia observed in women. By the way, Yoon et al. reported high PP increased the odds ratio of anemia. The mechanism of this relationship between PP and anemia remains equivocal. They suggested low whole blood volume relating to anemia can partially reduce DBP [34]. Therefore, female patients with CKD with higher DBP will decrease the incidence of anemia. Additionally, the variance in anemia risk factors between genders can be attributed to a confluence of cultural and social factors, including disparities in treatment recommendations or perceptions of the disease, as well as biological factors, including hormonal and genetic influences. Further research is recommended to investigate the mechanism underlying the sex-related disparities associated with anemia in CKD.

Figure 1 illustrates the distinct patterns of anemia risk factors at different stages of CKD. In early-stage CKD, a higher waist circumference showed an inverse relationship with anemia. In late-stage CKD, factors such as elevated HbA1c, diastolic blood pressure (DBP), and uric acid levels were associated negatively with anemia. In a previous cross-sectional study, an inverse relationship was observed between both overweight/obesity and central obesity with anemia, particularly noting that women with central obesity displayed a reduced likelihood of experiencing anemia [43]. Furthermore, Gillum et al. also reported a positive association between waist-to-hip ratio and serum ferritin concentration [44]. Similarly, Navaneethan et al. (2012) reported a negative association between high waist circumference and anemia among CKD patients, consistent with our findings. However, the exact mechanisms underlying this relationship remain unclear, and further research is needed to elucidate these pathways [45]. Previous epidemiological and clinical investigations have revealed a substantial positive correlation between HbA1c concentrations and hemoglobin levels, even after adjusting for age, gender, and race or ethnicity [46]. The absolute levels of HbA1c were significantly lower in both mild and moderate-severe anemia groups in comparison to the non-anemic group. Furthermore, a positive correlation was identified between HbA1c levels and key hematological parameters, including hemoglobin, ferritin, and red blood cell count [47]. The findings of this study revealed an inverse correlation between anemia and higher uric acid levels in individuals with late CKD. This suggests that a higher uric acid level could potentially indicate a better nutritional status [48]. Similarly, Su et al. (2016) observed a significant positive association between SUA levels and hematological indicators, including RBC count and hemoglobin, particularly in women [49]. However, this relationship remains debated. For instance, Guo et al. (2020) found that serum uric acid levels were negatively associated with hemoglobin and eGFR among CKD patients [50]. These conflicting findings highlight the need for further investigation into the underlying mechanisms of this relationship. Understanding these mechanisms may provide insights into the role of uric acid in CKD-related anemia and guide potential clinical interventions.

Nutritional indicators are important factors influencing anemia. Our univariate analysis revealed an association between lower BMI and anemia. However, when a multivariate logistic regression model was applied using stepwise selection, BMI was not retained in the final model. Recent research on kidney transplantation has assessed nutritional status using various indicators, including serum albumin, BMI, Onodera’s prognostic nutritional index, and the nutritional risk index [51]. The results indicate that BMI alone is not a reliable measure for differentiating study groups and does not effectively reflect malnutrition risk in the examined patients [51]. Additionally, factors such as nutritional profiles, substrate balance, and nutritional interventions may impact CKD outcomes [52]. A recent study emphasizes the importance of a comprehensive nutritional assessment for CKD patients [52]. This assessment should include a review of medical history, documentation of unintentional weight loss or a decline in physical function before hospitalization or ICU admission, a physical examination, and an evaluation of body composition, muscle mass, and strength [52].

The current study has several notable strengths. First, two large cohort data sources—the Epidemiology and Risk Factors Surveillance database and the NHIRD—were used to investigate the risk factors for anemia in patients with CKD. These databases provide comprehensive data across various domains encompassing patients’ demographic characteristics, biochemical data, lifestyle factors, medical and pharmacy claims data, diagnoses, and health service records. Second, the study elucidated differences in the risk of anemia related to sex and the severity of CKD. The application of 10-fold cross-validation for model selection and internal validation enhanced the reliability and robustness of our findings. Third, demographic characteristics and health-related behaviors were meticulously collected through face-to-face interviews conducted by well-trained interviewers, ensuring high-quality data were obtained.

This study also has certain limitations. First, this study may be subject to selection bias, given that patient recruitment for the Epidemiology and Risk Factors Surveillance Program was based on voluntary participation. This potential selection bias may affect the generalizability of our results to the broader population with CKD. Additionally, our study included patients from 14 hospitals and community-based programs, whereas nationwide CKD epidemiological studies in Taiwan have included a broader population. This difference in recruitment methodology likely contributed to the lower proportion of early-stage CKD and the higher proportion of advanced CKD in our cohort, as hospital-based cohorts tend to capture patients with more severe disease, compared to the nationwide study in Taiwan [53]. While this selection bias may limit direct comparisons with population-based CKD studies, our cohort remains representative of patients actively seeking medical care for CKD, making our findings clinically relevant. Furthermore, the distribution of CKD Stage 3 in our study was comparable to nationwide estimates, suggesting that our cohort includes a substantial proportion of patients at a critical stage of disease progression. Nevertheless, we acknowledge this limitation and recommend that future studies incorporate broader, population-based samples to further validate our findings. Second, our study adopted an observational cohort design rather than a randomized controlled trial. Consequently, unmeasured potential confounding variables could have influenced the results. Moreover, our study identifies associations between various factors and anemia but does not establish causality. Given the retrospective nature of our study, bidirectional relationships may exist; for example, anemia could be both a cause and a consequence of cardiovascular disease. Future research using longitudinal data and causal inference methodologies is warranted to further explore these complex relationships. Third, to ensure data integrity and minimize measurement error, we excluded participants with incomplete kidney function data, short follow-up durations, a single serum creatinine measurement, or no CKD diagnosis. While this approach enhanced the reliability of our dataset, it may have reduced statistical power and introduced selection bias. Although multiple imputation is commonly used to address missing data, prior research has shown that rounding imputed values can lead to biased parameter estimates, potentially affecting the validity of statistical conclusions [54]. Given these concerns, we opted not to perform multiple imputations in our analysis. Fourth, while our study identified a protective association between receiving low-sodium diet education and a reduced risk of anemia, we did not assess patient adherence to dietary recommendations or measure actual sodium intake. As a result, it remains unclear whether the observed effect is due to actual dietary modifications or other unmeasured factors. Future studies should incorporate objective measures of sodium intake and adherence to dietary recommendations to further validate this association. Fifth, in this study, anemia was defined using Taiwan’s National Health Insurance Research Database (NHIRD), requiring at least two outpatient or inpatient records of anemia diagnosis within one year. We acknowledge that this definition is based on clinical diagnosis rather than laboratory-confirmed hemoglobin values, which may impact case identification and comparability with other studies. This reliance on administrative coding could lead to potential misclassification and limit direct comparisons with research using standard clinical definitions, such as WHO or KDIGO hemoglobin thresholds. Sixth, our study observed an inverse association between elevated diastolic blood pressure (DBP) and anemia risk in CKD patients. However, we did not specifically account for the effects of antihypertensive therapies, which could influence both DBP and anemia risk. Future research should incorporate detailed medication data to better elucidate the impact of specific antihypertensive treatments on this association.

## 5. Conclusions

In conclusion, this study demonstrated that gout and cardiovascular disease were significantly associated with anemia in patients with CKD. However, this study also found that elevated DBP and receiving education on a low-sodium diet were inversely associated with anemia. Notably, the research highlighted differences in the risk factors for anemia in CKD related to sex and the severity of CKD. The findings of this research may aid clinicians in identifying patients with CKD who are at a higher risk of developing anemia, facilitating targeted screening initiatives for this particular patient group.

## Figures and Tables

**Table 1:** Characteristics of CKD patients according to anemia status (*n* = 5656).

Characteristic	Overall		Anemia				*p*-Value
			Without (*n* = 5137)		With (*n* = 519)		
	*n*/Mean	%/SD	*n*/Mean	%/SD	*n*/Mean	%/SD	
Age (years)	62.57	14.00	62.18	14.01	66.52	13.32	<0.0001
Sex, %							0.0001
Female	2354	41.62	2096	40.8	258	49.71	
Male	3302	58.38	3041	59.2	261	50.29	
Retire	3525	62.99	3153	62.02	372	72.66	<0.0001
Receive nutrition education	2939	52.17	2619	51.18	320	61.9	<0.0001
Receive low-sodium diet education	1700	30.17	1519	29.69	181	35.01	0.0138
Receive low phosphorus diet education	1242	22.04	1097	21.44	145	28.05	0.0007
Receive low-protein diet education	2032	36.07	1795	35.08	237	45.84	<0.0001
Physical examination							
Height (cm)	161.54	8.33	161.69	8.34	160.01	8.08	<0.0001
Weight (kg)	66.10	12.95	66.52	12.99	61.90	11.69	<0.0001
Waist (cm)	87.04	10.71	87.22	10.58	85.21	11.75	0.0012
SBP (mmHg)	132.30	17.24	132.39	17.32	131.39	16.44	0.2405
DBP (mmHg)	76.42	11.57	76.66	11.60	74.01	11.01	<0.0001
BMI (kg/m2)	25.27	4.14	25.38	4.14	24.13	3.91	<0.0001
Baseline eGFR (ml/min per 1.73 m^2^)	46.73	26.06	48.10	25.94	33.08	23.20	<0.0001
BUN (mg/dL)	28.61	18.11	27.76	17.67	36.58	20.20	<0.0001
Serum creatinine (mg/dL)	1.96	1.65	1.88	1.57	2.74	2.12	<0.0001
Total cholesterol (mg/dL)	185.35	42.70	186.40	42.71	175.09	41.24	<0.0001
Triglyceride (mg/dL)	139.16	78.77	140.54	79.27	125.68	72.39	<0.0001
Na (Sodium) (mmol/L)	139.36	3.43	139.41	3.39	138.99	3.72	0.0371
P (Phosphorus) (mg/dL)	3.87	0.88	3.85	0.88	4.00	0.82	0.0020
Uric acid (mg/dL)	6.88	1.77	6.87	1.76	6.98	1.86	0.1807
HbA1c (%)	6.79	1.51	6.82	1.52	6.51	1.45	0.0003
Hemoglobin/Hb (g/dL)	12.49	2.62	12.68	2.59	10.77	2.26	<0.0001
Proteinuria							0.0106
None	1512	42.57	1391	43.08	121	37.46	
Trace	437	12.3	406	12.57	31	9.6	
≥1+	1603	45.13	1432	44.35	171	52.94	
Health-related behaviors, %							
Cigarette smoking	1434	25.71	1341	26.5	93	18.02	<0.0001
Alcohol consumption	598	10.74	556	11.0	42	8.16	0.0558
Comorbidities, %							
Hypertension	3661	64.73	3302	64.28	359	69.17	0.0296
Diabetes mellitus	2392	42.29	2177	42.38	215	41.43	0.7097
Dyslipidemia	1395	24.66	1270	24.72	125	24.08	0.7888
Gout	997	17.63	885	17.23	112	21.58	0.0156
Ischemic heart disease	1356	23.97	1194	23.24	162	31.21	<0.0001
Stroke	1000	17.68	892	17.36	108	20.81	0.0574
Congestive heart failure	476	8.42	391	7.61	85	16.38	<0.0001
Urinary Tract Infection	1024	18.1	901	17.54	123	23.7	0.0006
Depression	398	7.04	346	6.74	52	10.02	0.0070
Cancer	942	16.65	817	15.9	125	24.08	<0.0001

Abbreviations: BMI, body mass index; SBP, systolic blood pressure; DBP, diastolic blood pressure.

**Table 2:** Predictive risk factors associated with anemia among CKD patients.

Characteristic	Anemia			*p*-Value
	OR	95%CI		
Receive low-sodium diet education	0.66	0.446	0.975	0.0368
DBP (mmHg)	0.98	0.965	0.999	0.0372
Gout	1.86	1.175	2.937	0.0081
Congestive heart failure	1.85	1.131	3.028	0.0143
C statistic				0.78
The 10-fold cross-validation accuracy rate				0.92

Adjusted: age, sex, serum creatinine, hemoglobin. Abbreviations: DBP, diastolic blood pressure.

## Data Availability

The datasets presented in this article are not readily available because they were obtained from the Taiwan National Health Insurance Research Database (NHIRD) and require approval for access. Requests to access the datasets should be directed to the Ministry of Health and Welfare, Taiwan (NHIRD_MOHW).

## Supplementary Materials

The following supporting information can be downloaded at: https://www.mdpi.com/article/10.3390/jcm14093088/s1. Supplementary Table S1. ICD-9-CM, ICD-10-CM, and ATC Codes Used for Disease and Medication Identification; Supplementary Table S2. Drug use of CKD with anemia patients among the early and late CKD patients (n = 519); Supplementary Table S3. Characteristics of CKD patients according to sex; Supplementary Table S4. Characteristics of among the early and late CKD patients; Supplementary Table S5. Univariate analyses of associations between predictive risk factors and anemia.
