# Supplementary material for: Sex- and Stage-Specific Predictors of Anemia in Chronic Kidney Disease: A Retrospective Cohort Study
Source: J Clin Med. 2025 Apr 29;14(9):3088. doi: 10.3390/jcm14093088 (PMC12072436; doi:10.3390/jcm14093088)
Supplement: Supplementary file 1 [file jcm-14-03088-s001.zip › jcm-3513830-supplementary.pdf]

Supplement Table S1. ICD-9-CM, ICD-10-CM, and ATC Codes Used for Disease and Medication Identification

Condition / Medication	ICD-9-CM	ICD-10-CM	ATC Codes (if applicable)
Anemia	280–289	D50–D89	
Erythropoietin (EPO)			B03XA01, B03XA02, B03XA03
Iron Supplements			A11BA, A13A, B03AA01, B03AA02, B03AA07, B03AB01, B03AB02, B03AB04, B03AB05, B03AC, B03AD01, B03AE02, B03AE03, B03AE04, B03AE10, V03AC02
Diabetes mellitus	250	E10–E14	
Hypertension	401–405	I10–I15	
Dyslipidemia	272	E7130, E752, E753, E755–E756, E77, E780–E789, E881, E882, E8889	
Gout	274	M10, M1A	
Cerebrovascular disease	430–438	I60-I69	
Ischemic Heart Disease	410–414, 557	I20–I25	
Congestive Heart Failure	398.91, 402.01, 402.11, 402.91, 404.01, 404.03, 404.11, 404.13, 404.91, 404.93, 425.4, 425.5, 425.7, 425.8, 425.9, 428	I0981, I110, I130, I132, I420, I422, I425, I426, I427, I428, I429, I43, I50	
Urinary Tract Infection	599.0	N39.0	
Depression	3004, 2962, 2963, 3090, 3091, 311	F341, F32, F33, F4321, F329	
Cancer	140–165, 170–195, 200–208, 2386, 196–199	Z51, C00, C02–C16, C7A, C18–C26, C30–C34, C37–C57, C60, C62–C86, C88,	

C96, D03, D3A,
D47Z9, E3122, J91,
R18

Supplement table S2. Drug use of CKD with anemia patients among the early and late CKD patients (n=519)

Drug use	CKD with anemia		Early CKD (n=142)		Late CKD (n=377)	
	n	%	n	%	n	%
None	124	23.89	72	50.70	52	13.79
Medicinal iron	98	18.88	49	34.51	49	13.00
Erythropoiesis-stimulating agents	65	12.52	4	2.82	61	16.18
Both Medicinal iron and Erythropoiesis-stimulating agents	232	44.7	17	11.97	215	57.03

Supplement table S3. Characteristics of CKD patients according to sex

Characteristic	Female (n= 2354)		Male (n= 3302)		p-value
	n/means	%/sd	n/means	%/sd	
Age (years)	61.69	13.49	63.21	14.32	<.0001
Retire	1494	64.37	2031	62.02	0.0770
Receive nutrition education	1164	49.68	1775	53.93	0.0018
Receive low sodium diet education	671	28.64	1029	31.27	0.0367
Receive low phosphorus diet education	477	20.36	765	23.25	0.0110
Receive low protein diet education	765	32.65	1267	38.5	<.0001
Physical examination					
Height (cm)	155.11	5.99	166.08	6.56	<.0001
Weight (kg)	60.29	11.45	70.21	12.36	<.0001
Waist (cm)	83.63	11.09	89.43	9.74	<.0001
SBP (mmHg)	131.83	17.81	132.64	16.82	0.1106
DBP (mmHg)	75.94	11.38	76.76	11.70	0.0140
BMI (kg/m^2)	25.05	4.50	25.42	3.86	0.0031
Baseline eGFR (ml/min per 1.73 m^2)	48.18	28.92	45.69	23.76	0.0006
BUN (mg/dL)	29.20	19.65	28.20	16.95	0.0559
Serum creatinine (mg/dl)	1.84	1.71	2.04	1.60	<.0001
Total cholesterol (mg/dL)	191.97	43.44	180.50	41.50	<.0001
Triglyceride (mg/dL)	139.43	77.72	138.96	79.54	0.8361
Na (Sodium) (mmol/L)	139.37	3.75	139.35	3.21	0.8331
P (Phosphorus) (mg/dL)	4.15	0.78	3.70	0.89	<.0001
Uric acid (mg/dL)	6.49	1.78	7.15	1.72	<.0001
HbA1c (%)	6.83	1.56	6.76	1.48	0.1731
Hemoglobin/Hb (g/dl)	11.74	2.60	13.03	2.50	<.0001
Proteinuria, %					0.2389
None	649	42.92	863	42.3	
Trace	200	13.23	237	11.62	
≥1+	663	43.85	940	46.08	
Health-related behaviors, %					
Cigarette smoking	85	3.66	1349	41.47	<.0001
Alcohol consumption	60	2.58	538	16.57	<.0001
Comorbidities, %					
Hypertension	1503	63.85	2158	65.35	0.2543
Diabetes mellitus	1003	42.61	1389	42.07	0.7039
Dyslipidemia	642	27.27	753	22.8	0.0001

Gout	200	8.5	797	24.14	<.0001
Ischemic heart disease	441	18.73	915	27.71	<.0001
Stroke	338	14.36	662	20.05	<.0001
Congestive heart failure	187	7.94	289	8.75	0.3026
Urinary Tract Infection	634	26.93	390	11.81	<.0001
Depression	199	8.45	199	6.03	0.0005
Cancer	364	15.46	578	17.5	0.0460

Abbreviations: BMI, body mass index; SBP, systolic blood pressure; DBP, diastolic blood pressure.

Supplement table S4. Characteristics of among the early and late CKD patients.

Characteristic	Early-CKD (n= 2825)		Late-CKD (n= 2831)		p-value
	n/means	%/sd	n/means	%/sd	
Age (years)	59.11	14.34	66.03	12.75	<.0001
Sex, %					0.0007
Female	1239	43.86	1115	39.39	
Male	1586	56.14	1716	60.61	
Retire	1512	53.75	2013	72.33	<.0001
Receive nutrition education	907	32.17	2032	72.18	<.0001
Receive low sodium diet education	535	18.98	1165	41.39	<.0001
Receive low phosphorus diet education	331	11.74	911	32.36	<.0001
Receive low protein diet education	562	19.94	1470	52.22	<.0001
Physical examination					
Height (cm)	162.08	8.47	160.97	8.14	<.0001
Weight (kg)	67.01	13.17	65.16	12.65	<.0001
Waist (cm)	86.53	10.65	87.56	10.74	0.0016
SBP (mmHg)	130.73	16.95	133.92	17.38	<.0001
DBP (mmHg)	77.31	11.22	75.50	11.86	<.0001
BMI (kg/m^2)	25.43	4.12	25.09	4.15	0.0041
Baseline eGFR (ml/min per 1.73 m^2)	68.95	15.64	24.54	11.30	<.0001
BUN (mg/dL)	16.35	5.64	39.51	18.41	<.0001
Serum creatinine (mg/dl)	0.95	0.25	2.96	1.83	<.0001
Total cholesterol (mg/dL)	188.43	43.25	182.27	41.93	<.0001
Triglyceride (mg/dL)	134.59	76.14	143.68	81.04	<.0001
Na (Sodium) (mmol/L)	139.63	3.51	139.23	3.39	0.0019
P (Phosphorus) (mg/dL)	3.56	0.75	3.97	0.89	<.0001
Uric acid (mg/dL)	6.22	1.53	7.50	1.76	<.0001
HbA1c (%)	6.90	1.56	6.69	1.46	<.0001
Hemoglobin/Hb (g/dl)	13.65	2.43	11.48	2.35	<.0001
Proteinuria, %					<.0001
None	1051	52.37	461	29.84	
Trace	290	14.45	147	9.51	
≥1+	666	33.18	937	60.65	
Health-related behaviors, %					
Cigarette smoking	655	23.4	779	28.04	<.0001
Alcohol consumption	340	12.17	258	9.29	0.0005

Comorbidities, %					
Hypertension	1700	60.18	1961	69.27	<.0001
Diabetes mellitus	1149	40.67	1243	43.91	0.0149
Dyslipidemia	821	29.06	574	20.28	<.0001
Gout	352	12.46	645	22.78	<.0001
Ischemic heart disease	575	20.35	781	27.59	<.0001
Stroke	420	14.87	580	20.49	<.0001
Congestive heart failure	134	4.74	342	12.08	<.0001
Urinary Tract Infection	565	20.00	459	16.21	0.0002
Depression	211	7.47	187	6.61	0.2233
Cancer	401	14.19	541	19.11	<.0001

Abbreviations: BMI, body mass index; SBP, systolic blood pressure; DBP, diastolic blood pressure.

Supplement table S5. Univariate analyses of associations between predictive risk factors and anemia. (n=5656)

Characteristic	Anemia		
	OR	95%CI	
Age (years)	1.03	1.02	1.03
Sex			
Female	1	ref	
Male	0.79	0.62	0.99
CKD stages			
Early CKD	1	ref	
Late CKD	2.86	2.24	3.65
Retire	1.68	1.32	2.14
Receive nutrition education	1.55	1.24	1.94
Receive low sodium diet education	1.32	1.05	1.66
Receive low phosphorus diet education	1.52	1.20	1.94
Receive low protein diet education	1.71	1.38	2.13
Physical examination			
Height (cm)	0.98	0.97	0.99
Weight (kg)	0.97	0.96	0.98
Waist circumference (cm)	0.98	0.97	0.99
SBP (mmHg)	1.00	0.99	1.00
DBP (mmHg)	0.98	0.97	0.99
BMI (kg/m^2)	0.92	0.90	0.95
Baseline eGFR (ml/min per 1.73 m^2)	0.98	0.97	0.98
BUN (mg/dL)	1.02	1.02	1.03
Serum creatinine (mg/dl)	1.25	1.20	1.30
Total cholesterol (mg/dL)	0.99	0.99	1.00
Triglyceride (mg/dL)	1.00	1.00	1.00
Na (Sodium) (mmol/L)	0.97	0.94	1.00
P (Phosphorus) (mg/dL)	1.20	1.07	1.35
Uric acid (mg/dL)	1.04	0.98	1.09
HbA1c (%)	0.85	0.77	0.93
Hemoglobin/Hb (g/dl)	0.68	0.65	0.71
Proteinuria			
None	1	ref	
Trace	0.88	0.58	1.32
≥1+	1.37	1.08	1.75

Health-related behaviors, %			
Cigarette smoking	0.61	0.48	0.77
Alcohol consumption	0.71	0.51	0.99
Comorbidities, %			
Hypertension	1.25	1.03	1.52
Diabetes mellitus	0.96	0.80	1.16
Dyslipidemia	0.97	0.78	1.19
Gout	1.32	1.06	1.65
Ischemic heart disease	1.50	1.23	1.82
Stroke	1.25	1.00	1.56
Congestive heart failure	2.38	1.84	3.07
Urinary Tract Infection	1.46	1.18	1.81
Depression	1.54	1.14	2.10
Cancer	1.68	1.35	2.08

Abbreviations: BMI, body mass index; SBP, systolic blood pressure; DBP, diastolic blood pressure.
